# Eating habits and weight status in Finnish adolescents

**DOI:** 10.1017/S1368980019001447

**Published:** 2019-06-21

**Authors:** Jannina Viljakainen, Rejane Augusta de Oliveira Figueiredo, Heli Viljakainen, Eva Roos, Elisabete Weiderpass, Trine B Rounge

**Affiliations:** 1 Folkhälsan Research Center, Biomedicum 1, PO Box 63 (Haartmansgatan 8), 00014 University of Helsinki, Finland; 2 Faculty of Medicine, University of Helsinki, Helsinki, Finland; 3 Department of Food and Environmental Sciences, University of Helsinki, Helsinki, Finland; 4 Department of Research, Cancer Registry of Norway, Oslo, Norway; 5 Department of Community Medicine, University of Tromsø – The Artic University of Norway, Tromsø, Norway; 6 Department of Medical Epidemiology and Biostatistics, Karolinska Institutet, Stockholm, Sweden

**Keywords:** Eating habits, Adolescent, Breakfast, Dinner, Weight status

## Abstract

**Objective:**

To investigate the association between eating habits and weight status in adolescents in Finland.

**Design:**

Cross-sectional study.

**Setting:**

The Finnish Health in Teens (Fin-HIT) study is a cohort study conducted in adolescents attending third to sixth grade in 496 schools in forty-four municipalities in Southern, Middle and Northern Finland in 2011–2014.

**Participants:**

Analyses included 10 569 adolescents from the Fin-HIT study aged 9–14 years (5005 boys and 5564 girls). Adolescents were categorized by their eating habits: healthy eaters (44·1 %; *n* 4661), unhealthy eaters (12·3 %; *n* 1298), and fruit and vegetable avoiders (43·6 %; *n* 4610); and they were grouped into weight status: underweight (11·1 %), normal weight (73·6 %) and excess weight (15·3 %).

**Results:**

We found an increased risk of underweight in fruit and vegetable avoiders (OR = 1·28; 95 % CI 1·12, 1·46). An irregular breakfast pattern showed an inverse association with underweight (OR = 0·70; 95 % CI 0·59, 0·84) and an increased risk of excess weight (OR = 1·56; 95 % CI 1·37, 1·77) compared with a regular breakfast pattern. An irregular dinner pattern was inversely associated with underweight (OR = 0·83; 95 % CI 0·69, 0·99) compared with a regular dinner pattern.

**Conclusions:**

Avoiding fruits and vegetables and following irregular breakfast and dinner patterns were associated with underweight and excess weight in adolescents.

Childhood excess weight, which increases the risk of non-communicable diseases^(^ ^1^ ^)^, has risen over time^(^ ^2^ ^,^ ^3^ ^)^, while childhood underweight has decreased in most countries^(^ ^3^ ^)^. Between the years 1980 and 2013, the global prevalence of excess weight increased by 47 % in 2–19-year-olds^(^ ^2^ ^)^. Childhood excess weight is also prevalent in Finland, reaching about 24 % in 2–16-year-old children in 2014–2015^(^ ^4^ ^)^. Thus, tackling this public health problem is essential.

Childhood excess weight could be prevented by enhancing physical activity^(^ ^5^ ^,^ ^6^ ^)^ and promoting healthy eating^(^ ^7^ ^)^, such as increased consumption of fruits and vegetables^(^ ^7^ ^–^ ^9^ ^)^. In most countries, including Europe, adolescents eat less fruits and vegetables than the minimum 400 g/d recommended by the WHO and the FAO^(^ ^10^ ^)^. Adolescents also favour more energy-dense, nutrient-poor foods (including ultra-processed foods, sweets and soft drinks)^(^ ^11^ ^)^. A healthy diet, consisting of an adequate consumption of fruits, vegetables, poultry, fish, low-fat dairy products and whole grains, decreases the risk of excess weight in paediatric and adult populations, while an unhealthy diet that is high in red and processed meats, refined grains, starchy foods, sweets and high-fat dairy has been associated with excess weight^(^ ^12^ ^)^. An abundant consumption of specific unhealthy food items such as sweets and sugar-sweetened soft drinks has been related both to underweight^(^ ^13^ ^)^ and excess weight^(^ ^14^ ^,^ ^15^ ^)^.

An irregular breakfast pattern, including breakfast skipping, has been found to increase the risk of excess weight in children and adolescents in several international studies^(^ ^16^ ^–^ ^20^ ^)^. Szajewska and Ruszczynski^(^ ^21^ ^)^ conducted a meta-analysis that included 57 481 European children and adolescents, which further emphasized the association between breakfast skipping and excess weight. Breakfast patterns and weight status have also been studied in Finland, where a high BMI was linked to more frequent breakfast skipping in children and adolescents^(^ ^22^ ^,^ ^23^ ^)^. In turn, having a family meal^(^ ^24^ ^)^ or following a regular meal pattern^(^ ^25^ ^)^ was suggested to protect against excess weight. In addition, following a regular breakfast, school lunch and dinner every school day may promote a healthy lifestyle in children and adolescents^(^ ^22^ ^)^.

There is a need for an in-depth understanding of the ongoing obesity epidemic and similarly to plan strategies to promote healthy weight and healthy eating in children and adolescents more effectively. There is a scarcity of Finnish studies on eating habits and their association with weight status in adolescents^(^ ^15^ ^,^ ^26^ ^)^ and young adults^(^ ^27^ ^,^ ^28^ ^)^. Thus, the aim of the present study was to examine whether eating habits and breakfast, lunch and dinner patterns during school days are associated with weight status in 9–14-year-old adolescents in Finland.

## Materials and methods

The Finnish Health in Teens (Fin-HIT) study was conducted in children aged 9–14 years, henceforth denoted ‘adolescents’, attending the third to sixth grades in 496 schools in forty-four municipalities in Southern, Middle and Northern Finland in 2011–2014. This cross-sectional study included 10 569 adolescents who had information on weight status, eating habits and consumption of meals available at baseline.

In the Fin-HIT cohort, the overall response rate was 30 % as described in detail elsewhere^(^ ^29^ ^)^. The distribution of main sociodemographic characteristics (sex and language) is similar to that in the general Finnish paediatric population^(^ ^30^ ^,^ ^31^ ^)^. Adolescents and their parents signed a consent form to participate in the study. The study protocol was approved by the Coordinating Ethics Committee of the Hospital District of Helsinki and Uusimaa (169/13/03/00/10).

Data collection took place in schools, where participants answered a web-based questionnaire on an electronic tablet. The questionnaire for adolescents covered, among others, lifestyle factors, diet and eating-related health behaviour. Adolescents’ age, sex and language spoken at home were obtained from the consent form or from questionnaires, and were confirmed afterwards through linkage to the National Population Information System at the Population Register Centre. Further information is described elsewhere^(^ ^29^ ^)^.

### Anthropometric measurements

The adolescents had their height and weight measured in a standardized manner by trained fieldworkers while wearing light indoor clothing^(^ ^29^ ^)^. In summary, height was measured to the nearest 0·1 cm with a plastic stadiometer at the end of expiration; weight was measured to the nearest 0·01 kg using daily-calibrated digital scales. All measurements were repeated twice. The weight of indoor clothing was estimated by fourteen clothing items and deducted from the measured weight prior to the calculation of BMI ([weight (kg)]/[height (m)]^2^). For the analysis, BMI was then classified as weight status (underweight, normal weight, overweight and obese) according to age- and gender-specific International Obesity Task Force cut-offs^(^ ^32^ ^)^. The groups of overweight and obese were combined and henceforth denoted as ‘excess weight’.

### Eating habits

We obtained information on eating habits with a fourteen-item FFQ related to the past month. The food items were key indicators of dietary fibre, calcium and products typical for youth culture^(^ ^33^ ^)^. The frequency of food consumption was self-reported and assessed by a 7-point scale varying from 0 (‘not consumed’) to 6 (‘consumed several times per day’).

Three eating habits were identified and defined in the Fin-HIT cohort as described elsewhere^(^ ^34^ ^)^. Briefly, factors were extracted from ten food items using factor analysis. The five resulting factors were used in *k*-means cluster analysis to detect the three different eating habits: ‘healthy eaters’, ‘unhealthy eaters’ and ‘fruit and vegetable avoiders’. Healthy eaters consumed more dark bread, fresh vegetables, fruits and berries compared with the others. Fruit and vegetable avoiders had the lowest intake of fresh vegetables, fruits and berries. Unhealthy eaters were the most frequent consumers of sweet pastries, biscuits or cookies, ice cream, sugary juice drinks, fast food (hamburgers or hot dogs) and salty snacks.

### Frequency of breakfast, lunch and dinner

Frequency of breakfast, lunch and dinner was asked with a question on ‘How often do you typically eat following meals during a school week?’ ranging from ‘never’ to ‘5 school days a week’ on a 6-point scale, which has been used previously in Health Behaviour in School-Aged Children (HBSC) Study^(^ ^33^ ^,^ ^35^ ^)^. Breakfast, lunch and dinner patterns were defined as regular when adolescents ate breakfast, lunch and dinner every school day and irregular if consumed less frequently.

### Statistical methods

The *χ* ^2^ test was used to investigate associations between weight status (underweight, normal weight and excess weight) and eating habits, breakfast patterns and dinner patterns. Multinomial logistic regression analysis was used to estimate the association between weight status and eating habits, breakfast and dinner patterns. OR and 95 % CI were calculated. The model was adjusted for adolescent’s age (as a quantitative variable), sex and language spoken at home (Finnish, Swedish or other). In our analysis, underweight and excess weight categories were compared with the normal weight category. Healthy eaters were the reference category and were compared with unhealthy eaters and fruit and vegetable avoiders. A regular breakfast pattern was the reference category and was compared with an irregular breakfast pattern. Similarly, a regular dinner pattern was the reference category and compared with an irregular dinner pattern. Interaction was tested using the likelihood ratio test. Interaction models were evaluated for: eating habits and sex; eating habits and breakfast patterns; eating habits and dinner patterns.

For all statistical analyses, we excluded in total 954 adolescents with missing values on weight status or missing information on eating habits or breakfast patterns or lunch patterns or dinner patterns.

The main statistical analyses were performed using the statistical software packages IBM SPSS Statistics version 24.0 and SAS version 9.4. A statistical significance level of 5 % was used.

## Results

In the final study sample, 47·4 % of adolescents were boys (*n* 5005) and 52·6 % were girls (n 5564); 11·1 % of adolescents were categorized as underweight, 73·6 % as normal weight and 15·3 % as excess weight. Compared with other categories, the underweight category had the highest percentage of girls (60·5 %). About 13·4 % of the participants were younger than 11 years old, 63·5 % were 11 years old and 23·1 % were older than 11 years. The majority (93·2 %) spoke Finnish at home. With respect to eating habits, 44·1 % of the study sample was categorized as healthy eaters, 12·3 % as unhealthy eaters and 43·6 % as fruit and vegetable avoiders (Table 1).

**Table 1:** Characteristics of the participants by weight status; adolescents aged 9–14 years (*n* 10 569) from the Finnish Health in Teens (Fin-HIT) study, 2011–2014

		Weight status						Total (*n*10 569; 100·0 %)		
		Underweight (*n* 1171; 11·1 %)		Normal weight (*n* 7784; 73·6 %)		Excess weight (*n* 1614; 15·3 %)					
		*n*	%	*n*	%	*n*	%	*n*	%	*P* value*
Age	>11 years	139	11·9	1065	13·7	211	13·1	1415	13·4	0·013
	11 years	748	63·9	4977	63·9	984	61·0	6709	63·5	
	<11 years	284	24·3	1742	22·4	419	26·0	2445	23·1	
Sex	Boy	463	39·5	3766	48·4	776	48·1	5005	47·4	<0·001
	Girl	708	60·5	4018	51·6	838	51·9	5564	52·6	
Language spoken at home	Finnish	1101	94·0	7249	93·1	1500	92·9	9850	93·2	0·010
	Swedish	42	3·6	348	4·5	55	3·4	445	4·2	
	Other	28	2·4	187	2·4	59	3·7	274	2·6	
Eating habits	Healthy eaters	489	41·8	3493	44·9	679	42·1	4661	44·1	0·009
	Unhealthy eaters	136	11·6	978	12·6	184	11·4	1298	12·3	
	Fruit and vegetable avoiders	546	46·6	3313	42·6	751	46·5	4610	43·6	
Breakfast pattern	Regular	1008	86·1	6368	81·8	1187	73·5	8563	81·0	<0·001
	Irregular	163	13·9	1416	18·2	427	26·5	2006	19·0	
Lunch pattern	Regular	1025	87·5	6825	87·7	1411	87·4	9261	87·6	0·955
	Irregular	146	12·5	959	12·3	203	12·6	1308	12·4	
Dinner pattern	Regular	1013	86·5	6526	83·8	1301	80·6	8840	83·6	<0·001
	Irregular	158	13·5	1258	16·2	313	19·4	1729	16·4	

* Results from the *χ* ^2^ test.

**Fig. 1:**
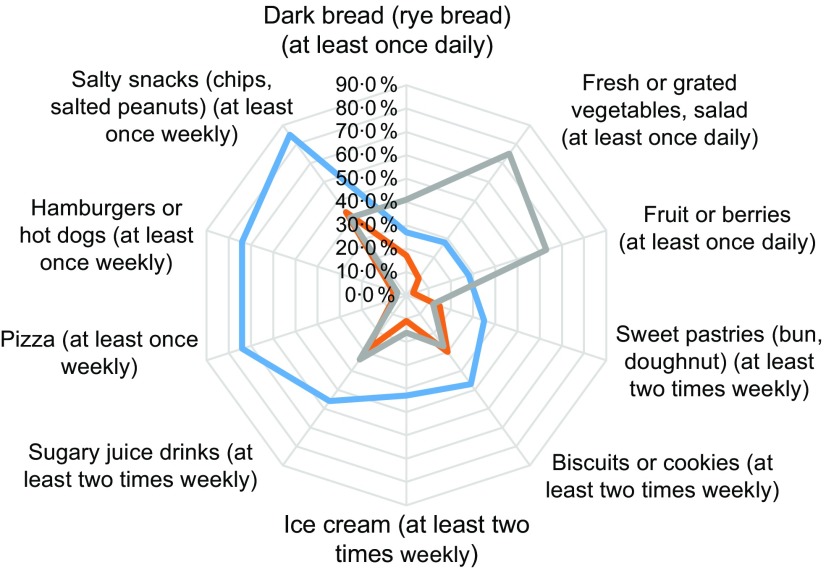
Frequency of high consumption of specified food items by unhealthy eaters (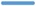, *n* 1298), fruit and vegetable avoiders (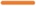, *n* 4610) and healthy eaters (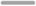, *n* 4661) among adolescents aged 9–14 years (*n* 10 569) from the Finnish Health in Teens (Fin-HIT) study, 2011–2014

Consumption of specific food items for these eating habits groups is described in detail in Fig. 1. Healthy eaters ate more dark bread, fresh vegetables, fruits and berries compared with fruit and vegetable avoiders and unhealthy eaters (Fig. 1). The fruit and vegetable avoiders consumed less fresh vegetables, fruits and berries compared with the other groups. Unhealthy eaters consumed more sweet pastries, sugary juice drinks and fast food (hamburgers or hot dogs), among other food items.

The normal weight category had a higher percentage of healthy eaters (44·9 %) compared with underweight (41·8 %) and excess weight categories (42·1 %, *P* = 0·009; Table 1). The underweight and excess weight categories had a higher percentage of adolescents in the fruit and vegetable avoiders group (46·6 and 46·5 %, respectively) compared with the normal weight category (42·6 %). Differences between weight status were also seen according to breakfast and dinner patterns, with regular breakfast and dinner patterns being more frequent in the underweight category (86·1 and 86·5 %, respectively) compared with the normal weight and excess weight categories (*P* < 0·001).

Lunch patterns were not included in the multinomial logistic regression model since they did not differ between weight status (*P* = 0·955; Table 1). Fruit and vegetable avoiders had an increased risk of underweight (OR = 1·28; 95 % CI 1·12, 1·46) compared with healthy eaters. Interestingly, unhealthy eating habit was not associated with weight status. A decreased risk of underweight (OR = 0·70; 95 % CI 0·59, 0·84) and an increased risk of excess weight (OR = 1·56; 95 % CI 1·37, 1·77) were observed in an irregular breakfast pattern when compared with a regular breakfast pattern. On the other hand, an irregular dinner pattern was inversely associated with underweight (OR = 0·83; 95 % CI 0·69, 0·99) compared with a regular dinner pattern (Table 2).

**Table 2:** OR and 95 % CI for eating habits, breakfast patterns and dinner patterns related to weight status among adolescents aged 9–14 years (*n* 10 569) from the Finnish Health in Teens (Fin-HIT) study, 2011–2014

Model 1	Weight status*											
	Normal		Underweight†					Excess weight†				
	*n*	%	*n*	%	OR	95 % CI	*P*value	*n*	%	OR	95 % CI	*P* value
Eating habits												
Healthy eaters	3493	44·9	489	41·8	1·00	–	–	679	42·1	1·00	–	–
Unhealthy eaters	978	12·6	136	11·6	1·16	0·95, 1·43	0·154	184	11·4	0·90	0·75, 1·08	0·243
Fruit and vegetable avoiders	3313	42·6	546	46·6	1·28	1·12, 1·46	<0·001	751	46·5	1·11	0·99, 1·24	0·087
Breakfast pattern												
Regular	6368	81·8	1008	86·1	1·00	–	–	1187	73·5	1·00	–	–
Irregular	1416	18·2	163	13·9	0·70	0·59, 0·84	<0·001	427	26·5	1·56	1·37, 1·77	<0·001
Dinner pattern												
Regular	6526	83·8	1013	86·5	1·00	–	–	1301	80·6	1·00	–	–
Irregular	1258	16·2	158	13·5	0·83	0·69, 0·99	0·047	313	19·4	1·09	0·94, 1·26	0·239

* Variables in the models: adolescents’ age, sex, language spoken at home, eating habits, breakfast patterns and dinner patterns.

† Reference category: normal weight.

We observed a significant interaction between eating habits and dinner patterns (*P* = 0·049). Among adolescents with a regular dinner pattern, fruit and vegetable avoiders had an increased risk of underweight (OR = 1·27; 95 % CI 1·10, 1·46) and excess weight (OR = 1·18; 95 % CI 1·04, 1·34) when compared with healthy eaters. Among adolescents with an irregular dinner pattern, unhealthy eaters were inversely associated with excess weight (OR = 0·58; 95 % CI 0·39, 0·87) when compared with healthy eaters (Table 3).

**Table 3:** OR and 95 % CI for eating habits related to weight status separately by dinner patterns* among adolescents aged 9–14 years (*n* 10 569) from the Finnish Health in Teens (Fin-HIT) study, 2011–2014

Model 2	Weight status†											
	Normal		Underweight‡					Excess weight‡				
	*n*	%	*n*	%	OR	95 % CI	*P* value	*n*	%	OR	95 % CI	*P* value
Model for regular dinner pattern†												
Eating habits												
Healthy eaters	3097	47·5	445	43·9	1·00	–	–	562	43·2	1·00	–	–
Unhealthy eaters	756	11·6	111	11·0	1·15	0·92, 1·45	0·217	144	11·1	0·99	0·81, 1·21	0·930
Fruit and vegetable avoiders	2673	41·0	457	45·1	1·27	1·10, 1·46	0·001	595	45·7	1·18	1·04, 1·34	0·010
Model for irregular dinner pattern†												
Eating habits												
Healthy eaters	396	31·5	44	27·8	1·00	–	–	117	37·4	1·00	–	–
Unhealthy eaters	222	17·6	25	15·8	1·30	0·76, 2·22	0·336	40	12·8	0·58	0·39, 0·87	0·009
Fruit and vegetable avoiders	640	50·9	89	56·3	1·41	0·95, 2·08	0·085	156	49·8	0·78	0·60, 1·03	0·083

* Likelihood ratio test to evaluate models with and without interaction between eating habit group and dinner patterns: *P* = 0·049.

† Variables in the models: adolescents’ age, sex, language spoken at home, eating habits and breakfast patterns.

‡ Reference category: normal weight.

There was no evidence of interaction between sex and eating habits (*P* = 0·108), and no interaction effects between eating habits and breakfast patterns (*P* = 0·094).

## Discussion

We found that adolescents who avoided fruits and vegetables were more likely to be underweight compared with healthy eaters. Adolescents with a regular dinner pattern, who also avoided fruits and vegetables, had an increased risk both of underweight and excess weight when compared with healthy eaters, thus emphasizing the importance of these habits. Unhealthy eating was not associated with weight status in our adolescents. An irregular breakfast pattern was associated with a higher risk of excess weight, but a lower risk of underweight compared with adolescents with a regular breakfast pattern. In addition, an irregular dinner pattern in adolescents was associated with a lower risk of underweight when compared with those with a regular dinner pattern. A subgroup of adolescents with a combination of irregular dinner pattern and unhealthy eating habit had a decreased risk of excess weight when compared with healthy eaters.

We found that the combination of avoiding fruits and vegetables and having a regular dinner was related to underweight. Our findings are comparable to those of a Norwegian study, in which the ‘unhealthy’ cluster was inversely associated with weight status. In that study, unhealthy eating habits consisted of, among other things, an inadequate fruit and vegetable intake and frequent consumption of snacks and soda^(^ ^13^ ^)^. However, the aforementioned study considered weight status as a continuous variable^(^ ^13^ ^)^, whereas in our study it was a categorical variable.

In the present study, we had limited information on the adolescents’ diet due to the shortness of the FFQ that was adapted to the age group, which may hamper the description of diet especially among fruit and vegetable avoiders. One possible explanation for higher risk of underweight among fruit and vegetable avoiders may be that they were picky eaters who restricted their vegetable and fruit intakes, but also had a little variety in other food consumption^(^ ^36^ ^)^. Picky eating has been associated with an increased risk of underweight and a lower weight status in a systematic review^(^ ^37^ ^)^, which is in accordance with our result. Underweight among fruit and vegetables avoiders could be due to a small portion size and limited energy intake, but we could not address these with our FFQ.

Fruit and vegetable avoiders with a regular dinner pattern had a higher risk of excess weight in adolescence. Similar results were found in another study among Finnish girls reporting that a low consumption of fruits and vegetables, and infrequent intake of junk food (i.e. pizza, candies or potato chips) were associated with excess weight. In that study, the group had other unhealthy behaviours as well, such as high screen time^(^ ^26^ ^)^. Unfortunately, we did not include information on other lifestyle factors, which could be meaningful here. Avoiding fruits and vegetable seems a potential indicator of weight status in adolescents.

In our study, unhealthy eating was not linked to weight status. Results from a Norwegian study^(^ ^38^ ^)^ support our findings, as they did not observe any association between consumption of unhealthy food items (i.e. junk food, sugar-sweetened soft drinks, biscuits) and risk of being overweight. On the contrary, an Italian study that included 58 928 adolescents observed an inverse association between excess weight and unhealthy food items (i.e. sweets, crisps and soft drinks)^(^ ^39^ ^)^.

Unhealthy eating habit and an irregular dinner pattern may be related because of snacking, although the evidence is inconsistent. One earlier study^(^ ^40^ ^)^ reported that replacing meals with unhealthy snacks and extra foods was not associated with weight status in American adolescents. However, more recent studies in American adolescents^(^ ^41^ ^)^ and Finnish men aged 17–21 years^(^ ^27^ ^)^ reported associations between snacking and a lower weight. We propose that unhealthy eaters and those with an irregular dinner pattern still had a moderate daily intake of energy that allowed them to maintain a healthy weight and protected them from underweight.

Previous international^(^ ^16^ ^–^ ^20^ ^)^ and Finnish^(^ ^22^ ^,^ ^23^ ^)^ studies have established associations between skipping breakfast and excess weight in children and adolescents, and our findings are in line with these. Besides this, we noticed that an irregular breakfast pattern protected against underweight. Our findings are unique in that sense; indeed, an Australian study^(^ ^42^ ^)^ demonstrated that skipping breakfast was associated with a higher risk of underweight. However, in that study^(^ ^42^ ^)^ skipping breakfast was defined by an energy intake less than 210 kJ (50 kcal) and was evaluated only at two time points (two 24 h recalls) in 2–17-year-old participants. We defined breakfast pattern as regular when consumed every school day and irregular if consumed less frequently. The mechanisms that explain how an irregular breakfast pattern affects underweight remain uncertain. Moreover, an irregular breakfast pattern might be related to other unhealthy behaviours that lead to weight gain, such as sedentary lifestyle as demonstrated previously^(^ ^23^ ^)^.

To our knowledge, the inverse association of an irregular dinner pattern with underweight has not been reported before. Moreover, skipping a main meal has been shown to be related to lower dietary quality^(^ ^43^ ^)^. We also observed that unhealthy eaters with an irregular dinner pattern were less likely to belong to the excess weight category. A previous Finnish study^(^ ^22^ ^)^ on children supports our findings on the association between an irregular dinner pattern and a higher weight status. A careful interpretation of these findings is warranted, since we had few adolescents (*n* 40) in this subgroup. Usually children eat dinner at home, which emphasizes the role of parents and the home environment. Child’s food intake, parental feeding behaviour^(^ ^44^ ^)^ together with parenting practices^(^ ^45^ ^)^ have earlier been associated with child’s weight status. It is possible that adolescents with the irregular dinner pattern have a home environment that stimulates behaviours related to underweight. Irregular eaters may eat something else that they do not consider as a meal.

The strengths of our study are a large nationwide cohort, which allowed us to study the current eating habits, breakfast and dinner patterns, and their associations both with underweight and excess weight in adolescents. Available data permitted us to examine potential confounders and modifiers for these associations. Furthermore, the data allowed us to evaluate the interaction between eating habits and dinner patterns in adolescents, which is a novel finding in the current study.

Although an earlier study^(^ ^46^ ^)^ showed that a short FFQ was a reliable tool for this age group, one limitation of our study is that we are unaware of the age group’s ability to accurately complete questionnaires. Moreover, the food items included in our FFQ were key indicators to assess both healthy and unhealthy diet among adolescents as suggested by the HBSC Study protocol^(^ ^33^ ^)^. It can be debated whether adolescents who belonged to the fruit and vegetable avoider group were also avoiding some other food items that our FFQ did not capture. Another limitation was the low response rate but, as shown, the sociodemographic characteristics were similarly distributed as in the Finnish paediatric population^(^ ^30^ ^,^ ^31^ ^)^. We had a cross-sectional study design; thus, we cannot conclude any causal relationship between eating habits and weight status based on our findings.

In our study, we were not able to record total energy intake and thus we could not adjust for this variable in the analysis. In addition, overweight and obese adolescents under-report their dietary intake more frequently, while underweight adolescents tend to over-report^(^ ^47^ ^)^. Hence, a reporting bias may have influenced our findings.

## Conclusion

In conclusion, fruit and vegetable avoiders had a higher risk of underweight, while adolescents with an irregular breakfast pattern had an increased risk of excess weight. Those who ate breakfast and dinner irregularly were less likely to be underweight. Future research is needed to study the causal relationship between eating habits and weight status among adolescents using a longitudinal design. Our findings can be used to help plan future interventions and guide public health professionals working with adolescents.
